# Engineering Approaches in Plant Molecular Farming for Global Health

**DOI:** 10.3390/vaccines9111270

**Published:** 2021-11-03

**Authors:** Advaita Acarya Singh, Priyen Pillay, Tsepo Lebiletsa Tsekoa

**Affiliations:** Future Production, Chemicals Cluster, Council for Scientific and Industrial Research, Pretoria 0001, South Africa; ASingh4@csir.co.za (A.A.S.); PPillay3@csir.co.za (P.P.)

**Keywords:** plant-made pharmaceutical, molecular farming, vaccine, glycoengineering, tyrosine sulfation, proteases inactivation

## Abstract

Since the demonstration of the first plant-produced proteins of medical interest, there has been significant growth and interest in the field of plant molecular farming, with plants now being considered a viable production platform for vaccines. Despite this interest and development by a few biopharmaceutical companies, plant molecular farming is yet to be embraced by ‘big pharma’. The plant system offers a faster alternative, which is a potentially more cost-effective and scalable platform for the mass production of highly complex protein vaccines, owing to the high degree of similarity between the plant and mammalian secretory pathway. Here, we identify and address bottlenecks in the use of plants for vaccine manufacturing and discuss engineering approaches that demonstrate both the utility and versatility of the plant production system as a viable biomanufacturing platform for global health. Strategies for improving the yields and quality of plant-produced vaccines, as well as the incorporation of authentic posttranslational modifications that are essential to the functionality of these highly complex protein vaccines, will also be discussed. Case-by-case examples are considered for improving the production of functional protein-based vaccines. The combination of all these strategies provides a basis for the use of cutting-edge genome editing technology to create a general plant chassis with reduced host cell proteins, which is optimised for high-level protein production of vaccines with the correct posttranslational modifications.

## 1. Introduction

Vaccination has been the most effective intervention in reducing death and morbidity resulting from infectious diseases in the last century. Vaccines stimulate humoral and cellular immunity in humans, thereby providing protection against infectious disease [1]. Several vaccines are protein-based; however, extracting these proteins from their natural sources may pose risks and may not be scalable. Inexpensive and scalable methods of production of recombinant protein are highly desirable. Traditional recombinant protein production approaches used are microbial fermentation, mammalian and insect cell culture, and transgenic animals. These systems have their drawbacks concerning upfront capital costs, scalability, and recombinant protein safety and authenticity [2,3,4].

Plant molecular farming (PMF) refers to the use of plants for the production of recombinant proteins that are of pharmaceutical or industrial interest. The first plant-produced protein of pharmaceutical interest was human growth hormone, produced in transgenic tobacco in 1986 [5]. The plant-based production of the first antibody was demonstrated in tobacco in 1989 [6]. The plant system offers a faster alternative and potentially more cost-effective and scalable platform for the mass production of proteins of pharmaceutical interest. The system also has a low risk of contamination compared with insect and mammalian cell culture production systems [7,8,9]. The high degree of similarity between the plant and mammalian secretory pathways makes it possible to efficiently produce highly complex protein-based vaccines [10]. Plant-based production has thus gained significant interest and is gaining wider acceptance [11,12].

**Figure 1:**
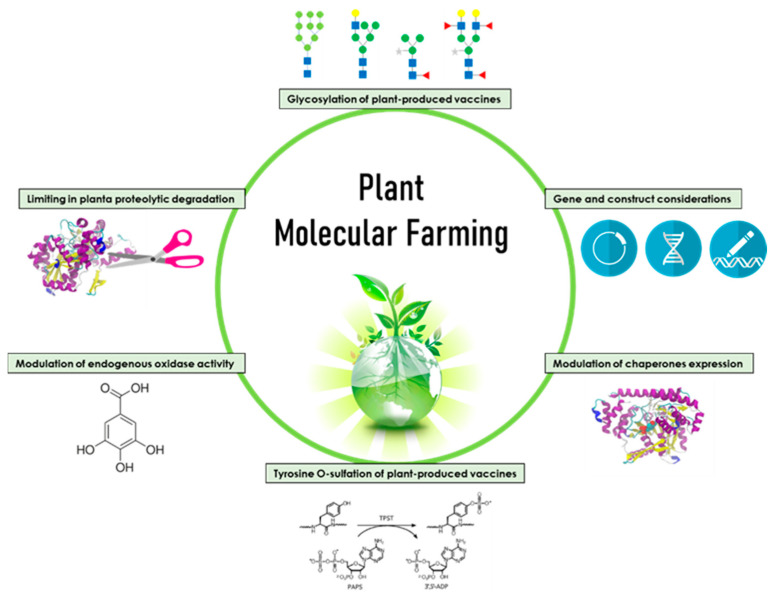
Engineering approaches in plant molecular farming.

Numerous plant species repurposed as recombinant protein production platforms include tobacco, potato, tomato, alfalfa, safflower, carrot, lettuce, strawberry, moss, duckweed, maize, wheat, and rice. Consolidation of the “molecular farming community” around the use of tobacco (Nicotiana tabacum) and its close relative Nicotiana benthamiana (*N. benthamiana*) as the most widely used species for protein expression has been seen recently [13]. These plant systems may be utilised as whole plants or in organ cultures (such as hairy roots) and cell suspension cultures. Various expression strategies have been exploited, such as (i) transgenic or transplastomic lines that are highly scalable; (ii) the use of transient expression in non-transgenic plants for rapid manufacturing; (iii) constitutive vs. tissue-specific vs. inducible expression; and (iv) different protein-targeting strategies to control product accumulation or post-translational modification for the facilitation of downstream processing [14,15,16]. Plants have proven versatile in producing a range of proteins, including proteins for nutritional supplements, industrial enzymes, and proteins of pharmaceutical interest [17,18]. Some examples of the many pharmaceutical proteins of interest are protein subunits, virus-like particles, monoclonal antibodies, cytokines, and human serum albumin. Despite the advantages of plant-production systems, some challenges may prevent the system from competing with the more conventional protein-based vaccine production systems, such as the folding of highly complex proteins and the processing of unique posttranslational modifications. The ability to tailor the plant system through cellular engineering techniques or process modification (Figure 1) to address these challenges is discussed in this review, demonstrating the versatility of the system and its ability to meet the demands of vaccine producers.

## 2. Improving the Yields of Recombinant Protein Vaccines

### 2.1. Gene and Construct Considerations

One of the desired outcomes in molecular farming is to achieve a high-expression yield of recombinant protein. To achieve high-expression yields of recombinant proteins, expression constructs must be optimized at all stages, from transcript to protein stability. Recently, it has been suggested that guanine-cytosine (GC) content of the gene of interest does not affect recombinant protein accumulation alone. Another important driver is codon–anticodon kinetics, which may be a major driver for translation efficacy [19]. Codon bias can result in a gene from one species being poorly expressed in another host organism as a result of the presence of unfavourable codons [20,21,22,23,24]. Correlations between the use of host-specific codon usage and the accumulation of correctly folded, active-recombinant protein should be identified. The yield of recombinant protein can therefore be improved through codon harmonisation, which involves replacing messenger ribonucleic acid (mRNA) codons with those that are preferred by the host [25].

Plant expression vectors are typically chimeric structures that have been constructed from repurposed plant viruses, such as tobacco mosaic virus (TMV), cowpea mosaic virus (CPMV), alfalfa mosaic virus, and potato virus X (PVX) [26]. The latest virus-based vectors are the deconstructed vectors that have non-essential genes of the virus replaced with the coding sequences of the genes of interest or those that drive high levels of translation. Viral vectors are used because of their ability to replicate at high quantities. The most well-known transient expression system, the MagnICON system, relies on constructs with a TMV and a PVX backbone [27]. A popular alternative is the pEAQ-HT expression system, which is based on the CPMV and a variant that improved plant transient expression though the rational design of synthetic 5′ and 3′ untranslated regions, giving rise to a pHREAC expression system [28,29,30]. Cloned recombinant genes within these constructs are bordered by various regulatory elements. Two elements important for high levels of transcription are the promoter and the polyadenylation site, which are often derived from the 19S and 35S transcripts of the cauliflower mosaic virus (CaMV) [31,32]. The CaMV 35S promoter has been the most popular choice for dicotyledonous plants (dicots), which are a strong constitutive promoter of the derived RNA transcripts. The arising RNA transcripts will be capped at its 5′ end by the cellular machinery, which is essential for translation in synergy with the poly (A) tail. The activity of the CaMV 35S promoter can be enhanced by duplicating the enhancer region [33]. However, this promoter produces lower activity in monocotyledonous plants (monocots), for which a preferred alternative is the maize ubiquitin-1 promoter [34]. The most widely used polyadenylation site is that of the CaMV 35S transcript, the *Agrobacterium tumefaciens* nos gene, and the pea ssu gene. Sequence-dependent RNA degradation or silencing can reduce the yield of recombinant protein [35]. This can be prevented through the coexpression of the recombinant protein along with a viral silencing suppressor, several of which have been demonstrated transiently in *N. benthamiana* [36]. The p19 suppressor from tomato bushy stunt virus (TBSV) is perhaps the most well studied and functions by binding siRNA and prevents RISC assembly, thereby increasing recombinant protein yields [37].

### 2.2. Modulation of Chaperone Expression

Chaperones mediate the folding of proteins within the endoplasmic reticulum (ER). These chaperones provide stringent quality control and ensure that misfolded protein is targeted for endoplasmic reticulum-associated degradation (ERAD) [38]. Recombinant protein expression stresses the machinery, and by doing so, induces the unfolded protein response (UPR), which results in an increase in chaperone expression. The modulations of selected chaperones can therefore be used as a strategy to improve recombinant protein production. However, the alteration in the expression of these chaperones may also have an influence of the phenotype of the plant and the endogenous protein levels [39]. A slight increase above normal expression levels of binding protein 1 (BiP1) in transgenic rice increased the recombinant protein yield in rice seeds [39]. Subtle modulation of the ER-associated folding pathway was shown to increase recombinant protein yields; however, an alternative is the coexpression of the protein with a chaperone from another host [25]. Increases in yields in *N. benthamiana* were observed for several human viral glycoproteins destined for use as vaccine antigens in the presence of human calreticulin [25]. The coexpression of human calreticulin with HIV gp140 expression also resulted in an increase in accumulated yield of recombinant protein [40].

### 2.3. Modulation of Endogenous Oxidase Activity

The extraction of recombinantly produced protein in plants requires homogenisation of green leaf material; this process also results in the release of phenolic compounds. Polyphenol oxidases (PPOs) catalyse the formation of covalent complexes between the recombinant protein and the phenol compounds. This can result in the aggregation and precipitation of recombinant protein, significantly reducing yield and quality [41]. Reductions in phenolic oxidation can be achieved through the addition of antioxidant in the extraction buffer; however, this in turn can potentially increase the complexity of the extraction and the overall cost of the production process, affecting its techno-economic viability [42]. The adoption of ribonucleic acid interference (RNAi) targeting PPO has been shown to reduce the browning process of potato tubers and apples [43]. The clustered regularly interspaced short palindromic repeats (CRISPR)-associated, nuclease (CRISPR/Cas)-mediated knock out of a single member of the potato PO gene family also demonstrated a reduction in PPO activity by 69% without any phenotypic effects [44]. A similar approach can be adopted in *Nicotiana* spp.; however, since PPOs function in defence, elimination of PPO activity may interfere with normal plant growth and phenotypic properties.

### 2.4. Limiting in Planta Proteolytic Degradation

Proteases are triggered by developmental and environmental cues and function in a housekeeping role, activation of zymogens, removal of non-functional protein, and pathogen defence [45,46,47,48]. The targeting of recombinant proteins through the plant secretory pathway for post-translational modifications (PTMs), such as glycosylation and sulfation, makes them susceptible to proteases, which are auto-catalytically mature in low-pH environments [49,50]. The MEROPS database (28/04/2020) lists 515 known or putative peptidases and 98 non-peptidase homologues in *N. benthamiana* to date, which are possibly responsible for in planta degradation of some recombinantly produced proteins [51]. A major problem encountered with the plant-based production of protein in *Nicotiana* species is the in planta proteolytic degradation of some recombinantly produced proteins [52,53]. Proteolytic degradation is not only limited to in planta degradation during protein expression, ex planta degradation is also possible during extraction and downstream processing [53,54,55,56,57]. Proteolytic degradation may not only reduce the purity and yields of recombinantly produced protein but can also compromise the structural integrity of these proteins. Proteolytic degradation such as this can result in altered biological activity or no protein production at all, ultimately resulting in a bottleneck in the production of biopharmaceuticals [58,59,60]. Agrobacterium infiltration of plants such as *N. benthamiana* has been found to elicit an immune response, the hypersensitive response, which includes the induction of pathogenesis-related (PR) genes and the accumulation of extracellular PR proteins [61,62,63]. These responses not only reduce subsequent infection but may also hinder agrobacterium-mediated transgene delivery [64,65,66,67]. In conjunction with the hindrance of agrobacterium-mediated transgene delivery, the upregulation of these PR proteins causes the degradation of recombinantly produced protein and triggers the process of senescence, which results in the degradation of host cellular protein. Degradation of the chloroplastic protein occurs in the initial stages of senescence, resulting in reduced photosynthetic capacity of the leaf [68]. The downregulation of proteases has been shown to have beneficial effects on the accumulation of recombinantly produced protein [69]. Initial cleavage of recombinant protein is targeted to sterically exposed segments [70]. Reducing the in planta activities of these proteases could improve both the quality and yield of the produced recombinant protein [70].

Commonly employed strategies involve the use of ribonucleic acid interference (RNAi), or either the coexpression of companion protease inhibitors or proton channels, which have been previously used to create protease activity-depleted or inactive environments [56,69,71,72]. RNAi-targeted silencing of proteases has been demonstrated in whole-plant and cell suspension culture. Attempts were made to stably and transiently silence the CysP gene in whole tobacco plants [69]. Transient silencing of CysP6 was demonstrated to be the more successful strategy, which increased in planta accumulation of the recombinant protein of interest, interleukin (IL)-10, in comparison with transgenic silencing [69]. Protease silencing attempts in suspension cultures of cell lines producing the 2F5 antibody showed decreased protease degradation with increased accumulation of the antibody [56]. RNAi silencing of proteases has proven successful, alternatively, the coexpression of plant-derived protease inhibitors, such as cystatins, cysteine protease inhibitors are also possible [71,72]. Transgenic tobacco plants constitutively expressing the rice cysteine protease inhibitor oryzacytatin-I have significantly lower cysteine protease activity when compared with non-transgenic plants [71,73]. This lower cysteine protease activity has been shown to increase the production of recombinant protein [71]. Transiently expressing these protease inhibitors, such as tomato cystatin SICYS8 in the secretory pathways of *N. benthamiana,* has been shown to prevent degradation of recombinant protein [72]. Regulation of the pH in this pathway has been shown to reduce in planta proteolytic degradation of the recombinantly produced protein. A strategy for increasing the Golgi lumen pH through the coexpression of an influenza virus M2 proton channel successfully stabilised acid-labile recombinant proteins and peptides in leaf cells [74].

## 3. Posttranslational Modifications of Plant-Produced Vaccines

### 3.1. Glycosylation of Plant-Produced Vaccines

Many protein-based vaccines require glycosylation, yet the role of the glycosylating glycan may vary in functionality. Glycosylation in eukaryotic cells occurs in proteins that transit via the ER and Golgi secretory pathway [75]. Glycosylation occurs in three forms, namely: N-glycosylation on Asn residues, O-glycosylation on Ser/Thr or hydroxylated Lys and Pro residues, and C-glycosylation on Trp residues [75]. N-glycosylation is a major protein glycosylation type, influencing folding, trafficking, protein interactions, and efficacy of subunit vaccines and other biologics.

An example of this is the glycosylation of the Fc region of antibodies (Abs) and its significant impact on antibody effector functions, such as antibody-dependent, cell-mediated cytotoxicity (ADCC) and antibody-dependent, cell-mediated virus inactivation (ADCVI) [76,77,78]. The initial stages of N-glycosylation and associated glycan processing occurs in the endoplasmic reticulum, a process that is conserved between mammals and plants. Further processing steps to form complex N-glycans occurs in the Golgi apparatus through a process that differs between mammals and plants. Plants lack the ability to produce highly complex N-glycans owing to fewer glycosyltransferases, which elongate the N-glycans. Plants also produce non-human glycans that contain β1,2-xylose and/or α1,3-fucose residues [79], and these glycans have effects on the pharmacokinetics of a biopharmaceutical product, as well as immunogenicity in humans [80,81]. *N. benthamiana* has been glycoengineered by either the downregulation or elimination of xylosyl- and fucosyltransferase with RNAi and CRISPR/Cas9, respectively [82]. Production in this engineered host resulted in the transient production of mAbs with a predominantly mammalian GnGn glycan structure [80,81]. The resulting glycoforms are more homogenous than FDA-approved mAbs produced in mammalian cell cultures [80,81].

Plants lack the ability to produce complex N-glycans; to augment this, the glycosyltransferases and the entire pathway for the synthesis, transport, and transfer of the glycosylating residues must be introduced into the plants. Plants also lack the ability to perform the β1,4-linked galactose (galactosylated) modification and to cap with sialic acid (N-acetylneuraminic acid, sialylated) residues. The transient and stable introduction of pathways have been developed to enable the incorporation of human-type-N-glycans with sialic acid onto glycoproteins [83,84,85]. The coordinated expression of both mammalian sialic acid pathways and human polysialyltransferases results in the formation of polysialic acid-containing glycoproteins with similar characteristics to their mammalian cell culture-derived variants [86].

Despite the ability to introduce human-like glycosylation, the ability to fully glycosylate available glycosylation sites in recombinant proteins remains a challenge. Members of the CAP256-VRC26 lineage of HIV antibodies have been produced in *N. benthamiana* (ΔXTFT), achieving approximately 50% glycosylation on the plant-produced Fc regions consistent with reports of other plant-produced Abs [87,88]. Incomplete glycosylation of transiently plant-produced proteins has been reported frequently [80,89,90]; however, close to complete glycosylation was achieved with plant-produced VRC01 [91]. However, this is dependent on the expression host; the extent of sequon occupancy has been observed to differ between plant species. Plant oligosaccharyltransferase (OST) complexes are made of multiple subunits; however, the composition and function of each is poorly understood [92,93]. As such, the cause of differences is still unknown. However, differences can arise from the inability of plants’ OST complex to recognise the N-glycosylation site, resulting in incomplete glycosylation of the plant-produced glycoproteins. A viable approach to increase the in planta N-glycosylation efficiency is through the coexpression of OST subunits [87]. The transient expression of a single subunit of the Leishmania major (L. major) OST complex resulted in increased N-glycan occupancy in various immunoglobulin structures [87].

Unlike with N-glycans, less research focus has been placed on O-glycans, the second major type of glycosylation that is catalysed within the secretory pathway [94]. O-glycans are significant as O-glycans present on viral envelope glycoprotein, which are potential vaccine candidates [95]. This modification is catalysed by N-acetylgalactosaminyltransferases, which plants lack, along with the glycotransferases that are responsible for the elongation and branching of these O-glycans [96]. These mammalian-type O-glycans can be generated de novo [96].

### 3.2. Tyrosine O-Sulfation of Plant-Produced Vaccines

Tyrosine sulfation is essential for protein–protein interactions, especially for antibodies (Abs) that target the V1V2 region of human immunodeficiency virus (HIV), such as PG9 and the CAP256-VRC26 lineage [90,97,98]. Antibodies play a pivotal role in possible vaccination strategies for pre-exposure prophylaxis against HIV. Functionality of V1V2 targeting mAbs requires an O-sulfated tyrosine in the CDR H3 loop of the antigen-binding domain to facilitate tight binding of the gp120 envelope glycoprotein in a manner that mimics the HIV-1 gp120 affinity for the sulfated CCR5 [99]. Tyrosine sulfation is catalysed by a tyrosylprotein sulfotransferase (TPST) that is localised to the subcellular trans-Golgi network, thereby limiting sulfation to proteins that transit through the secretory pathway (ER and Golgi). In plants, a putative TPST gene was identified by Komori and co-workers in Arabidopsis [100,101]. *N. benthamiana* is commonly used for Ab production; however, attempts to produce PG9 in planta resulted in inactive Ab due to the lack of tyrosine O-sulfation. This suggests that, unlike Arabidopsis, *N. benthamiana* lacks the necessary machinery to carry out tyrosine sulfation [102]. Previous attempts to find a TPST candidate in the *N. benthamiana* draft genome were unsuccessful [90,103]. Despite this, the expression of active V1V2 targeting antibodies, PG9, CAP256-VRC26.08, and CAP256-VRC26.09 in *N. benthamiana* was demonstrated. This was achieved through the transient coexpression of the mAbs with human TPST1 (hTPST1) engineered for post-Golgi targeting [88,90]. The coexpression of hTPST1 with CAP256-VRC26 antibodies produced both mono- and di-sulfated CAP256-VRC26 species and restored neutralising potency against a broad panel of HIV-1 strains [88].

## 4. Conclusions and Future Impact of Engineering Strategies in Plant Molecular Farming

During the last thirty years, great progress has been made in demonstrating the utility of plant production systems for producing protein-based vaccines. The utility of the plant production system was demonstrated during the coronavirus disease 2019 (COVID-19) pandemic, with vaccine candidates currently in various clinical trial phases. Two examples are the Medicago and GlaxoSmithKline coronavirus-like particle (CoVLP) (NCT04636697, Phase II/III) and the capsid virus-like particle (cVLP) from Kentucky Bioprocessing, Inc. (NCT04473690, Phase I/II). Various engineering approaches have been demonstrated to tailor-make authentic protein-based vaccines at sufficient yields. These approaches allow the engineering of the plant expression systems, enabling the production of increasingly complex protein molecules with correct PTMs. Obtaining correctly folded protein is of critical importance to protein function. The further exploration of recombinant protein and chaperone coexpression combinations is of great interest in optimising the host secretory pathway for increased recombinant protein yield. In so doing, the exploration of new frontiers of PTMs, such as the incorporation of O-glycan structures to viral envelope glycoproteins, is of potential medical interest.

*In planta* and *ex planta* oxidation and proteolytic degradation remain as much a hurdle to produce structurally correct and functional plant-produced intravenous vaccines as they do for edible vaccines. These deleterious processes are not limited to the extraction process during the downstream processing of plant-produced intravenous vaccines. An area of growing interest is the modulation of deleterious host cell proteins, which are not essential to plant growth and production of functional recombinant protein, in an attempt to both simplify the downstream processing and increase synthesis capacity. The development and optimisation of CRISPR/Cas9-based genome editing allows for precise modification of the plant genome, creating the basic chassis for tailoring the production of any protein-based vaccine or pharmaceutical. Improved capacity can be achieved through the diversion of plant cell molecular machinery from host cell protein to the production of the target recombinant protein. Reducing the abundance of host cell protein, such as ribulose-1,5-bisphosphate carboxylase/oxygenase (RuBisCO), which can account for 40% of the total soluble protein (TSP) in leaf cells, can facilitate the simpler downstream processing of the recombinant protein. In generating tailor-made plants to produce certain protein-based pharmaceuticals, it is important to consider introducing whole pathways to incorporate human-like PTMs specific to the recombinant protein of interest. These engineering prospects will promote future advances in the plant molecular farming field. Though the optimal utility of the plant system is yet to be realised, we hope that these developments will bring about more traction in attracting the adoption of plant molecular farming field for global health.

## Abbreviations

mRNA: TMV: PVX, CPMV, CaMV, TBSV, ER, ERAD, UPR, BiP1, PPOs, CRISPR/Cas9, PR, ADCC, ADCVI, OST, Abs, TPST, hTPST, TSP.
